# A DAQ-Device-Based Continuous Wave Near-Infrared Spectroscopy System for Measuring Human Functional Brain Activity

**DOI:** 10.1155/2014/107320

**Published:** 2014-08-10

**Authors:** Gang Xu, Xiaoli Li, Duan Li, Xiaomin Liu

**Affiliations:** ^1^State Key Laboratory of Cognitive Neuroscience and Learning and IDG/McGovern Institute for Brain Research, Beijing Normal University, Beijing 100875, China; ^2^Center for Collaboration and Innovation in Brain and Learning Sciences, Beijing Normal University, Beijing 100875, China; ^3^School of Information Science and Engineering, Yanshan University, Qinhuangdao 066004, China; ^4^School of Electronic Information and Control Engineering, Beijing University of Technology, Beijing 100124, China

## Abstract

In the last two decades, functional near-infrared spectroscopy (fNIRS) is getting more and more popular as a neuroimaging technique. The fNIRS instrument can be used to measure local hemodynamic response, which indirectly reflects the functional neural activities in human brain. In this study, an easily implemented way to establish DAQ-device-based fNIRS system was proposed. Basic instrumentation components (light sources driving, signal conditioning, sensors, and optical fiber) of the fNIRS system were described. The digital in-phase and quadrature demodulation method was applied in LabVIEW software to distinguish light sources from different emitters. The effectiveness of the custom-made system was verified by simultaneous measurement with a commercial instrument ETG-4000 during Valsalva maneuver experiment. The light intensity data acquired from two systems were highly correlated for lower wavelength (Pearson's correlation coefficient *r* = 0.92, *P* < 0.01) and higher wavelength (*r* = 0.84, *P* < 0.01). Further, another mental arithmetic experiment was implemented to detect neural activation in the prefrontal cortex. For 9 participants, significant cerebral activation was detected in 6 subjects (*P* < 0.05) for oxyhemoglobin and in 8 subjects (*P* < 0.01) for deoxyhemoglobin.

## 1. Introduction

Different neuroimaging methods measuring the electrophysiological (e.g., electroencephalography (EEG) and magnetoencephalography (MEG)) or metabolic (e.g., functional magnetic resonance imaging (fMRI) and positron emission tomography (PET)) aspects of neural activity have been widely used in physiology and psychology research. In these neuroimaging techniques, fMRI has a very high spatial resolution to measure the blood oxygen level-dependent (BOLD) signal that highly correlated with deoxyhemoglobin and becomes a gold standard for vivo imaging of brain activity [1, 2]. However, fMRI also has disadvantages, including high sensitivity to head motion, a loud and restrictive environment, low temporal resolution, and a very high cost, which limit its application in children or other special populations. Since Frans Jobsis first demonstrated the feasibility of monitoring the concentration change of oxyhemoglobin (oxy-Hb) and deoxyhemoglobin (deoxy-Hb) in 1977 [3], functional near-infrared spectroscopy (fNIRS) is getting more and more attentions in the past 20 years as an effective research and clinical tool [4–7]. Compared with fMRI, fNIRS has many advantages including portability, higher temporal resolution, lower cost, being less sensitive to head motion, and being capable of long time measurement, thus making it a more user-friendly neuroimaging method for both adults and infants [1, 8, 9].

The light in the near-infrared spectral range (650–950 nm) is able to penetrate human tissue, and few amounts of photons can be detected without being completely absorbed several centimeters away from the emitter on scalp. With well-chosen wavelength, the quantity of NIR light being absorbed reveals concentration change of hemoglobin in the brain tissue where photons propagate through. Based on different measuring principle, fNIRS instruments can be divided into three categories: continuous wave (CW), frequency domain (FD), and time domain (TD). TD and FD techniques offer the possibility of measuring absolute concentrations of hemoglobin, by obtaining absolute characterization of the tissue optical properties (scattering and absorption coefficients). However, quantification is not a crucial factor in neuroscience studies, because it is more important to statistically significantly detect a relative change than to quantify it in absolute terms [10]. Due to high cost and technology complexity, only one TD- and one FD-based commercial instrument are available in the market worldwide [6, 11]. CW-fNIRS instrument is absolutely dominant in the market with prices varying from some $10,000 for simple systems to several $100,000 for whole-head imaging systems [10]. Although the price is much lower than fMRI, commercial product is still very expensive. For the need of neuroimaging research by fNIRS, many laboratories have tried to build custom-made systems with more flexibility and lower cost [12–18].

For CW-fNIRS instrumentation, we can choose either embedded system or system based on data acquisition (DAQ) device as a candidate for hardware platform. Typically, an embedded system is housed on a single microprocessor board with dedicated function implemented. The portability of embedded system makes it possible to monitor hemodynamic response in living environment [13, 15, 16, 19–22]. But, due to smaller number of channels and limited sensor choice, this kind of system is more suitable for measuring signal on the non-hair-bearing forehead. DAQ device also provides a way to interface between signal and computer. Usually, DAQ hardware contains multiple components such as multiplexer, analog-to-digital converter (ADC), digital-to-analog converter (DAC), and high-speed timers, and the circuit has been optimized and calibrated for accurately measuring physical signals with minimal distortion. The DAQ-based fNIRS system could be designed and integrated with more flexibility and shortened development cycle [12, 14, 23–26]. It is a better choice to build a custom-made CW-fNIRS system by using a DAQ device, which provides user with a fast and flexible solution for instrumentation.

In this study, a single-channel CW-fNIRS system based on multifunction desktop DAQ device was introduced. In Section 2.1, four types of DAQ hardware device with varied characteristics were compared.  Section 2.2 described the details of the CW-fNIRS system, which contains source driving, signal conditioning, sensor selection, and optical fiber customization. The functionality of the software interface was described in Section 2.3, including source multiplexing and digital demodulation technique. To evaluate the effectiveness of the system, two separate experiments were implemented to assess relative change in regional cerebral hemodynamic response and functional neural activation during task period, as described in Section 3.

## 2. System Design

### 2.1. Hardware

**Figure 1:**
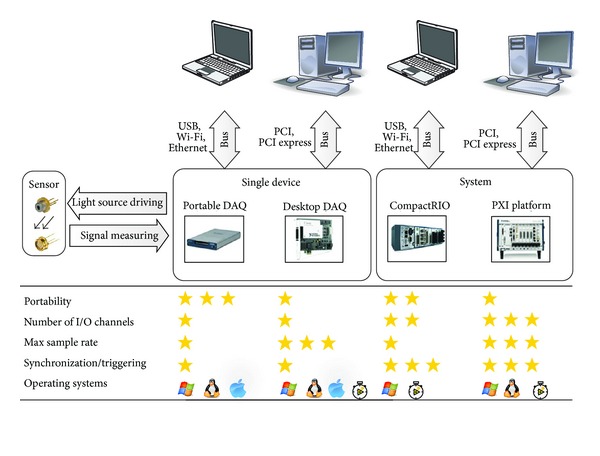
Characteristics of the DAQ hardware devices. Either single device or measuring system is eligible to drive a light source or to acquire signal from a detector. The features of these devices or platforms are varied. The star ratings from 1 to 3 indicate low, medium, and high level, respectively. The supported operating system contains Windows, Linux, Mac OS, and real-time operating system.

The DAQ devices can be classified into two categories, single device and real-time system. Each category contains two types of DAQ devices with diverse specifications, as illustrated in Figure 1. Single-device DAQ hardware includes portable and desktop ones. With the plug-and-play external bus of universal serial bus (USB), portable DAQ device is capable of being designed with specifics of low weight, battery-powered, and minimum size, while desktop DAQ device, which have to be installed into a PC slot, offers high-speed data streaming and deterministic data transfer, with a shared high bandwidth. However, poor real-time performance is a vital disadvantage for both types of single-device DAQ hardware. Because the user interface is running on the general-purpose operating system rather than a real-time operating system, the accuracy of timing will be variable depending on the workload of the operating system.

However, a real-time system, such as CompactRIO (real-time modular controller, National Instruments Corporation) or PXI (PCI eXtensions instrumentation platform, National Instruments Corporation), consisted of a chassis to control timing and synchronization, which give us the ability to prioritize tasks so that the most critical task can always take control of the processor when needed, thus guaranteeing reliable predictability and execution. Another advantage of the real-time system is that you can choose different unique-purpose input/output (I/O) modules, which makes the configuration more flexible.

### 2.2. System Description

**Figure 2:**
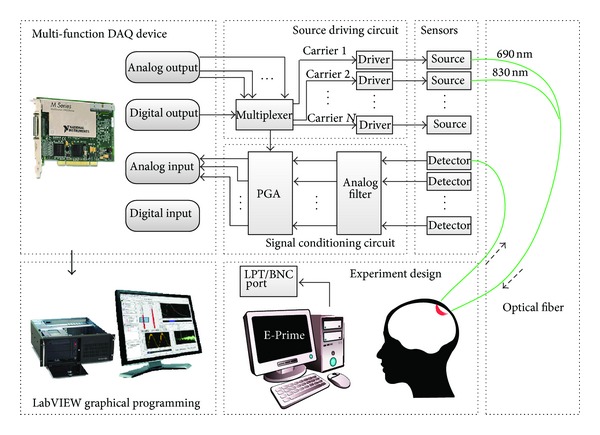
The schematic diagram of hardware for the custom-made CW-fNIRS system.

In the proposed system, a multifunction desktop DAQ device (PCI-6251, National Instruments Corporation) is used to provide basic physical I/O channels (analog input (AI), analog output (AO), digital input (DI), and digital output (DO)), by which we can drive the laser diodes or acquire optical signals. The architecture of the custom-made system is shown in Figure 2, and details of each part of the system are as follows.

#### 2.2.1. Source Driving Circuit

The frequency multiplexing technology is used to remove environmental stable interference sources like ambient light, power line, and 1/f noise generated by the electronics. Due to the limited DACs to generate carrier frequency, the time division multiplexing technique is also implemented to illuminate light sources for multichannel measurement. Each NIR light source is amplitude modulated at different carrier frequency, ranging from 2 to 4 kHz, with an interval of 200 Hz. The source driving circuit includes multiplex and laser drivers (iC-NZP, iC-Haus GmbH) for emitting NIR light in consecutive time-slot.

#### 2.2.2. Signal Conditioning Circuit

The scattered NIR light is collected and converted into electrical signal by photodiodes. Depending on many factors including source-detector distance, scalp thickness, and hair color and density, the light collected is 7–9 orders smaller in magnitude than that emitted at the source [16]. Due to the small light intensity, the output signal of detectors is generally filtered and amplified to increase signal-to-noise ratio (SNR) in the signal conditioning circuit. Second-order active band-pass Butterworth filter is applied in the analog circuit, with high-pass filter used to remove ambient light interference and electrical noise and low-pass filter used to prevent aliasing [25].

A programmable gain amplifier (PGA204, Texas Instruments Inc.) was used to meet the input voltage range of the ADC. The programmable gain of 1/10/100/1000 could be automatically determined according to the amplitude of the observed signal, by using transistor-transistor logic (TTL) levels. When receiving external trigger signal as a synchronizing marker, the DI channel will sample the TTL signal through a BNC or parallel port, which is generated by a computer for stimuli presentation or other neural imaging instruments.

#### 2.2.3. Source and Detector

Either laser diode (LD) or light emitting diode (LED) is eligible as a NIR light source. Based on stimulated emission, LD emits coherent light with a narrow bandwidth, which provides better monochromaticity [10]. Laser usually propagates by optical fiber to the skin to avoid possible injury of heat effect [27]. As a valid alternative source, LED is based on spontaneous emission and the incoherent light emits with a larger bandwidth. To gain more accurate results, a LD pair (HL6738MG/HL8338MG, Thorlabs Inc.) with wavelength of 690 nm and 830 nm was used in the customized system. Silicon photodiodes (SiPD), avalanche photodiode (APD), and photomultiplier tube (PMT) are frequently used photodetectors that convert light signal into electrical one. With the trade-off among sensitivity, gain, respond speed, and price, an APD module (Hamamatsu C5460-01, Hamamatsu Photonics K.K.) was used in the system. By integrating low-noise current-to-voltage amplifier, the APD module outputs voltage signal with a default gain of 30.

#### 2.2.4. Optical Fiber

The optical fiber contains three categories in materials: plastic optical fiber, glass optical fiber, and silica optical fiber. With the balance between quality and cost, silica and glass fiber are used for coupling light from skin to emitter and detector, respectively. Light emitted from the laser diode pair with both wavelengths is guided to the scalp through 3-meter-long multimode silica optical fibers, which combined into one bundle physically to ensure the same emitting location. A 3-meter-long glass fiber bundle collects the scattered light from the tissue and transfers it to the detector. The diameter of the fiber bundle for detector is 2.7 mm to ensure enough amount of light being collected.

### 2.3. Software Interface

**Figure 3:**
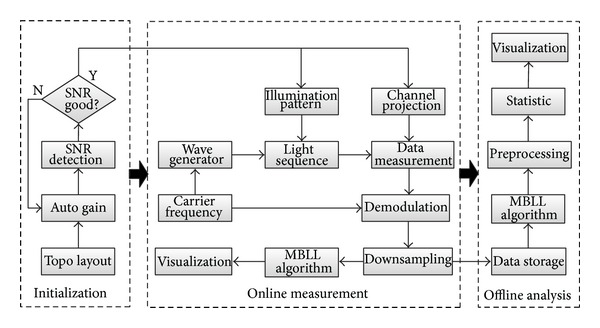
The schematic diagram of the software for the custom-made CW-fNIRS system.

With hundreds of math and signal processing functions, graphical programming software LabVIEW (National Instruments Corporation) is ideal for a measurement or control system based on DAQ devices. Prior to online measurement, initialization is needed to acquire signal with a higher SNR, and data will be analyzed offline after acquisition (Figure 3). With predefined topography layout, the physical I/O channels of the DAQ device can be projected to the measurement channels assigned by source detector pair. Automatic gain setting for each channel enables the amplitude of the signal meeting the input range of the ADC. If the SNR of the signal is not high enough in some channels, the corresponding optodes should be adjusted manually to improve the coupling to the skin.

To distinguish mixed light sources from multiple emitters around the detector, time and frequency multiplexing methods are commonly used in CW-fNIRS system. Time multiplexing provides a continuous time-slot sequence to illuminate light with different source or wavelength. Frequency multiplexing distinguishes each component from mixed light sources by hardware-based lock-in amplifier [12, 23, 28] or software-based method such as in-phase and quadrature (IQ) demodulation technique [25]. Compared with lock-in amplifier array, digital method is a much less expensive solution to demodulate signals. Although the digital demodulation for multichannel will increase the computation cost, sampling queues will not lose data by using the Producer/Consumer design pattern in LabVIEW.

Two sources with different wavelength were designed to be illuminated with 25-millisecond time-slot in the proposed system simultaneously. The continuous illuminating time-slot should be set larger than a threshold due to distortion of demodulated signal on the edge, which is related to sampling rate, carrier frequency, and digital filter response. The sampling rate is 62.5 KS/s for each channel which is higher than 10 times of maximum carrier frequency at least. By assigning the corresponding carrier frequency, the data can be demodulated by using IQ digital demodulator program. Then, after downsampling to 10 Hz, the data of light intensity was saved and converted to the relative change of oxy-Hb and deoxy-Hb by modified Beer-Lambert law (MBLL).

## 3. Experimental Evaluation

Two experiments were designed to verify the effectiveness of the custom-made fNIRS system. A Valsalva maneuver task was carried out to change the hemodynamic response in the brain, and data was recorded simultaneously with a commercial fNIRS instrument (ETG-4000, Hitachi, Ltd.) as a comparison. Another experiment of mental arithmetic task provided the evidence of cerebral neural activation to the mental workload.

### 3.1. Experimental Design

#### 3.1.1. Valsalva Maneuver

One volunteer attended the task of Valsalva maneuver, which is a clinical paradigm for testing hemodynamic response [29]. The purpose of this experiment was to compare the light intensity signals between commercial instrument and the custom-made system. The four optodes of two systems (each optode pair include one emitter and one detector) were placed side by side on the prefrontal around the FP1 position according to 10–20 international system. The distance between source and detector was fixed to 3 cm. The synchronous trigger signals were outputted from a computer to custom-made system and ETG-4000 via parallel port and serial port, respectively, as a marker. The subject was seated on a comfortable chair and instructed to perform the Valsalva maneuver. By plugging the nose, closing the mouth, and attempting to expire air, the hemodynamic response would change according to the brain blood pressure fluctuations. This block-designed paradigm contains 5 blocks, and each block includes 30 s task and 120 s rest period.

#### 3.1.2. Mental Arithmetic

In the Valsalva maneuver paradigm, the hemodynamic change is mainly attributed to extracerebral tissue (scalp and skin). To further evaluate the functional activation in cerebral cortex, another experiment of mental arithmetic task is performed, which is a well-established psychological paradigm for assessing mental workload. It is widely accepted that cortical activation could be indicated by an incremental rise in oxy-Hb accompanied by a decrease in deoxy-Hb [30–33]. Studies indicated that highly complex cerebral networks involved during arithmetic task include the prefrontal cortex, fusiform gyrus, cingulate cortex, cerebellum, insula, and the parietal cortex [34, 35]. Both ventrolateral prefrontal cortex (VLPFC) and dorsolateral prefrontal cortex (DLPFC) are considered as regions of interest related to mental arithmetic task [36, 37]. VLPFC is located on the inferior frontal gyrus, being attributed to the anatomical structures of Brodmann's area (BA) 47, 45, and 44. Left VLPFC has been proven to be related to the cognitive control of memory by neuropsychological and neuroimaging studies [38, 39].

**Figure 4:**
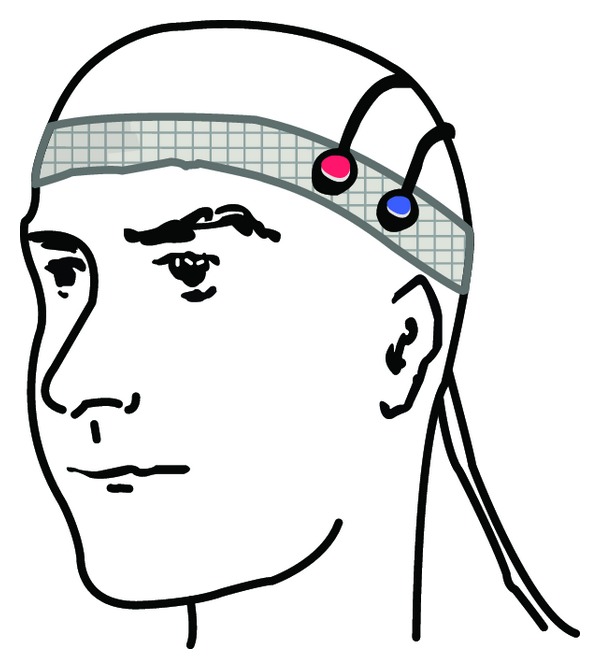
Positions of optodes for the source (red) and the detector (blue) in the mental arithmetic experiment.

Nine university-aged young volunteers (6 males, 3 females; age of 23.8 ± 3.8) were instructed to perform a 690-second-long mental arithmetic task while seated on a comfortable chair. As shown in Figure 4, two optodes (source and detector) were placed on the subject's left VLPFC area, located at F7 of the 10–20 system [40]. The optodes were secured to the head with a Velcro strap. The paradigm was block-designed and the stimuli were presented by using E-Prime v1.1 (Psychology Software Tools, Inc.). Each block was 60 s long which consists of 20 s task and 40 s rest period. The entire experiment contains 10 blocks and a 90 s prescan. During the task period, each subject was requested to solve a formula mentally, which is subtracting a 3-digit number from a 3-digit number (e.g., 523 − 276 = ?). During the rest period, participants were instructed to keep their eyes open with mental relaxation.

### 3.2. Data Analysis

Data analysis was performed in MATLAB 2011a (Mathworks Inc.) offline. In the Valsalva maneuver experiment, Pearson's linear correlation coefficient was calculated to compare the relationship of light intensity signals recorded using custom-made system and ETG-4000, with the same sampling rate of 10 Hz. Then oxy-Hb and deoxy-Hb were converted based on MBLL [41, 42], respectively, according to molar extinction coefficients of the sources. These hemodynamic signals were expressed as the product of the relative change of hemoglobin concentration and the effective optical path length (mM × mm). With band-pass filtering (0.004–2 Hz) of 3th order Butterworth filter, both oxy-Hb and deoxy-Hb were visually inspected for the entire time course.

In the mental arithmetic experiment, all the processing for oxy-Hb and deoxy-Hb was performed using NIRS_SPM (Bio Imaging Signal Processing lab, Korea), which is an open-source SPM and MATLAB-based software package for statistical analysis of fNIRS signals [43]. High-pass filter based on discrete cosine transform (DCT) with cut-off 128 s was implemented for detrending (about twofold duration of one block). The precoloring method [44] was used to remove the physiology noise (such as heart rate and respiration). To examine the hemodynamic performance of the task, statistical analysis based on general linear model (GLM) was implemented to evaluate the activation in prefrontal cortex. By statistical inference, a *t*-statistic value was calculated in the GLM approach to demonstrate the statistical power of brain activation.

### 3.3. Experimental Results

#### 3.3.1. Valsalva Maneuver

**Figure 5:**
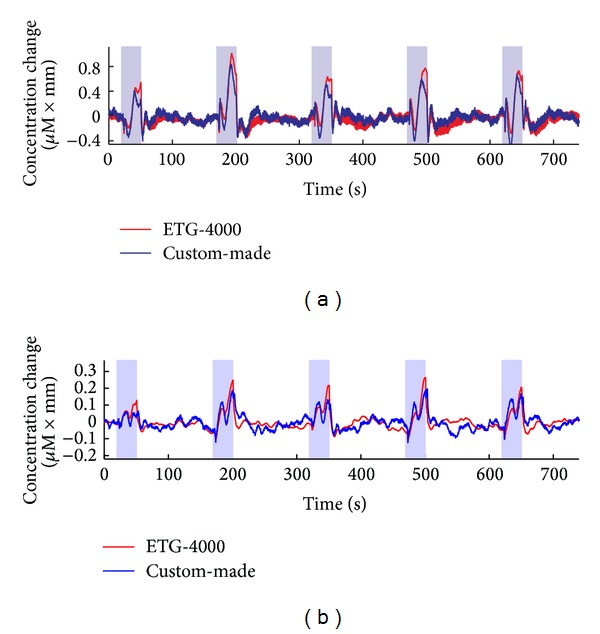
Comparison of time courses of oxy-Hb (a) and deoxy-Hb (b) acquired from ETG-4000 and custom-made system during Valsalva maneuver task.

By comparing signals from ETG-4000 and the proposed system, the light intensity was highly correlated for both lower wavelength (*r* = 0.92, *P* < 0.01) and higher wavelength (*r* = 0.84, *P* < 0.01). Relative change of oxy- and deoxyhemoglobin could be converted from light intensity by using MBLL. The band-pass filtered time courses of oxy-Hb and deoxy-Hb were shown in Figure 5. It could be clearly seen that both Oxy-Hb and deoxy-Hb derived from the ETG-4000 (in red) and custom-made (in blue) system presented higher correlation.

#### 3.3.2. Mental Arithmetic

**Figure 6:**
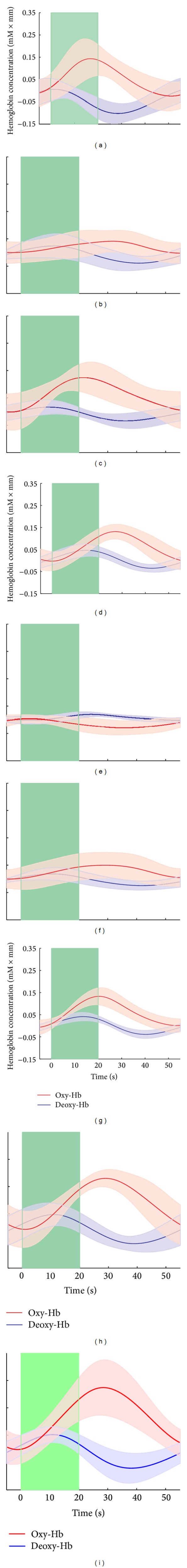
Block-averaged concentration change of oxy-Hb and deoxy-Hb for 9 subjects in the mental arithmetic experiment. The values were given in the form of mean (solid line) and standard deviation (colored shade area). The green shade area indicates the task period of mental arithmetic.

**Figure 7:**
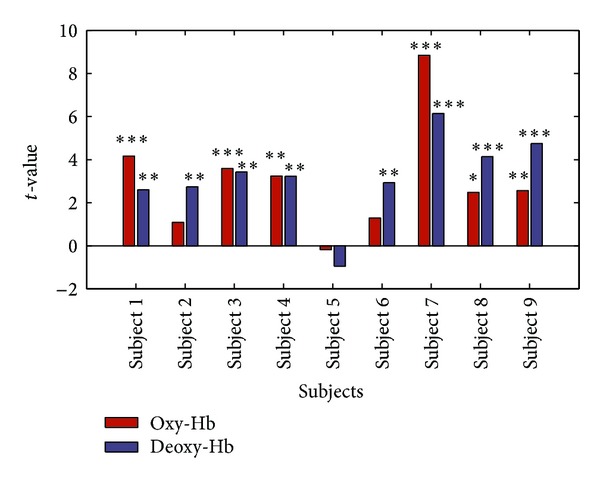
The *t*-values in GLM indicated cortical activation during mental arithmetic experiment for 9 subjects, respectively. The notations ∗∗∗, ∗∗, and ∗ indicate significant difference in the *t*-values at *P* < 0.001, *P* < 0.01, and *P* < 0.05, respectively.

The block-averaged time courses for both oxy-Hb and deoxy-Hb were shown in Figure 6. The data was baseline corrected by subtracting the mean intensity of 5 s preceding trial onset. By visual inspection, the activation of deoxy-Hb has a time delay of about 5–10 s compared with oxy-Hb in most cases. With statistical analysis based on GLM, the *t*-values for both oxy-Hb and deoxy-Hb were shown in Figure 7. Significant cerebral activation in oxy-Hb (*P* < 0.05) was detected in 6 of 9 participants and that in deoxy-Hb in 8 of 9 participants (*P* < 0.01). For both oxy-Hb and deoxy-Hb, activation failed to be detected for only 1 subject, probably due to the deviated position of measuring channel.

## 4. Conclusion

The DAQ device and graphical programming software LabVIEW provide us with an easy-to-build solution for CW-fNIRS system, with low price and customized specifications. To meet varied demands such as channel number, sampling rate, portability, modularity, and so forth, four types of DAQ products were compared. In this paper, a custom-made CW-fNIRS system based on desktop DAQ device was proposed, and detailed description was discussed including analog circuit design and frequency multiplexing technique. Two experiments were carried out to evaluate the effectiveness of the proposed system. Statistical results demonstrated that the custom-made system is capable of detecting cerebral neural activation in the cognitive related experiment. This single-channel system could expand to a multichannel one by simply multiplying sensor in the same hardware platform with minimal additional cost.

## References

[1] Cui X, Bray S, Bryant DM, Glover GH, Reiss AL (2011). A quantitative comparison of NIRS and fMRI across multiple cognitive tasks. *NeuroImage*.

[2] Shibasaki H (2008). Human brain mapping: hemodynamic response and electrophysiology. *Clinical Neurophysiology*.

[3] Jobsis FF (1977). Noninvasive, infrared monitoring of cerebral and myocardial oxygen sufficiency and circulatory parameters. *Science*.

[4] Lloyd-Fox S, Blasi A, Elwell CE (2010). Illuminating the developing brain: the past, present and future of functional near infrared spectroscopy. *Neuroscience and Biobehavioral Reviews*.

[5] Hoshi Y (2011). Towards the next generation of near-infrared spectroscopy. *Philosophical Transactions of the Royal Society A*.

[6] Ferrari M, Quaresima V (2012). A brief review on the history of human functional near-infrared spectroscopy (fNIRS) development and fields of application. *NeuroImage*.

[7] Quaresima V, Bisconti S, Ferrari M (2012). A brief review on the use of functional near-infrared spectroscopy (fNIRS) for language imaging studies in human newborns and adults. *Brain and Language*.

[8] Steinbrink J, Villringer A, Kempf F, Haux D, Boden S, Obrig H (2006). Illuminating the BOLD signal: combined fMRI-fNIRS studies. *Magnetic Resonance Imaging*.

[9] Vermeij A, van Beek AHEA, Olde Rikkert MGM, Claassen JAHR, Kessels RPC (2012). Effects of aging on cerebral oxygenation during working-memory performance: a functional near-infrared spectroscopy study. *PLoS ONE*.

[10] Scholkmann F, Kleiser S, Metz AJ (2014). A review on continuous wave functional near-infrared spectroscopy and imaging instrumentation and methodology. *NeuroImage*.

[11] Torricelli A, Contini D, Pifferi A (2014). Time domain functional NIRS imaging for human brain mapping. *NeuroImage*.

[12] Bauernfeind G, Leeb R, Wriessnegger SC, Pfurtscheller G (2008). Development, set-up and first results for a one-channel near-infrared spectroscopy system. *Biomedizinische Technik*.

[13] Muehlemann T, Haensse D, Wolf M (2008). Wireless miniaturized in-vivo near infrared imaging. *Optics Express*.

[14] Soraghan C, Matthews F, Markham C, Pearlmutter BA, O'Neill R, Ward TE (2008). A 12-channel, real-time near-infrared spectroscopy instrument for brain-computer interface applications. *EEE Engineering in Medicine and Biology Society*.

[15] Lareau E, Lesage F, Pouliot P, Nguyen D, Le Lan J, Sawan M (2011). Multichannel wearable system dedicated for simultaneous electroencephalographynear-infrared spectroscopy real-time data acquisitions. *Journal of Biomedical Optics*.

[16] Lareau E, Simard G, Lesage F, Nguyen D, Sawan M Near infrared spectrometer combined with multichannel EEG for functional brain imaging.

[17] Adhika DR, Hazrati MK, Hofmann UG (2012). An experimental setup for brain activity measurement based on near infrared spectroscopy. *Biomedical Technologies*.

[18] Sawan M, Salam MT, le Lan J (2013). Wireless recording systems: From noninvasive EEG-NIRS to invasive EEG devices. *IEEE Transactions on Biomedical Circuits and Systems*.

[19] Choi J, Choi M, Kim J, Bae H (2013). Efficient data extraction method for near-infrared spectroscopy (NIRS) systems with high spatial and temporal resolution. *IEEE Transactions on Biomedical Circuits and Systems*.

[20] Safaie J, Grebe R, Moghaddam HA, Wallois F (2013). Toward a fully integrated wireless wearable EEG-NIRS bimodal acquisition system. *Journal of Neural Engineering*.

[21] Atsumori H, Kiguchi M, Obata A (2009). Development of wearable optical topography system for mapping the prefrontal cortex activation. *Review of Scientific Instruments*.

[22] Ito T, Hirano T, Mitsui Y, Akiyama H, Ohgi S, Mizuike C Design of brain machine interface using portable Near-InfraRed Spectroscopy.

[23] Coyle SM, Ward TE, Markham CM (2007). Brain-computer interface using a simplified functional near-infrared spectroscopy system.. *Journal of neural engineering*.

[24] Nissilä I, Noponen T, Kotilahti K (2005). Instrumentation and calibration methods for the multichannel measurement of phase and amplitude in optical tomography. *Review of Scientific Instruments*.

[25] Joseph DK, Huppert TJ, Franceschini MA, Boas DA (2006). Diffuse optical tomography system to image brain activation with improved spatial resolution and validation with functional magnetic resonance imaging. *Applied Optics*.

[26] Piper SK, Krueger A, Koch SP (2014). A wearable multi-channel fNIRS system for brain imaging in freely moving subjects. *NeuroImage*.

[27] Orihuela-Espina F, Leff DR, James DRC, Darzi AW, Yang GZ (2010). Quality control and assurance in functional near infrared spectroscopy (fNIRS) experimentation. *Physics in Medicine and Biology*.

[28] Sato H, Kiguchi M, Kawaguchi F, Maki A (2004). Practicality of wavelength selection to improve signal-to-noise ratio in near-infrared spectroscopy. *NeuroImage*.

[29] Gao L, Elwellelwell CE, Kohl-Bareis M, LaManna JC, Puchowicz MA, Xu K, Harrison DK, Bruley DF (2011). Effects of assuming constant optical scattering on haemoglobin concentration measurements using NIRS during a valsalva manoeuvre. *Oxygen Transport to Tissue XXXII*.

[30] Gervain J, Mehler J, Werker JF (2011). Near-infrared spectroscopy: a report from the McDonnell infant methodology consortium. *Developmental Cognitive Neuroscience*.

[31] Perrey S (2008). Non-invasive NIR spectroscopy of human brain function during exercise. *Methods*.

[32] Perrey S, Thedon T, Rupp T (2010). NIRS in ergonomics: its application in industry for promotion of health and human performance at work. *International Journal of Industrial Ergonomics*.

[33] Mandrick K, Derosiere G, Dray G, Coulon D, Micallef J, Perrey S (2013). Utilizing slope method as an alternative data analysis for functional near-infrared spectroscopy-derived cerebral hemodynamic responses. *International Journal of Industrial Ergonomics*.

[34] Verner M, Herrmann MJ, Troche SJ, Roebers CM, Rammsayer TH (2013). Cortical oxygen consumption in mental arithmetic as a function of task difficulty: a near-infrared spectroscopy approach. *Frontiers in Human Neuroscience*.

[35] Arsalidou M, Taylor MJ (2011). Is 2+2=4? Meta-analyses of brain areas needed for numbers and calculations. *NeuroImage*.

[36] Pfurtscheller G, Bauernfeind G, Wriessnegger SC, Neuper C (2010). Focal frontal (de)oxyhemoglobin responses during simple arithmetic. *International Journal of Psychophysiology*.

[37] Kawashima R, Taira M, Okita K (2004). A functional MRI study of simple arithmetic—a comparison between children and adults. *Cognitive Brain Research*.

[38] Badre D, Wagner AD (2007). Left ventrolateral prefrontal cortex and the cognitive control of memory. *Neuropsychologia*.

[39] Brass M, Derrfuss J, Forstmann B, Von Cramon DY (2005). The role of the inferior frontal junction area in cognitive control. *Trends in Cognitive Sciences*.

[40] Yanagisawa K, Masui K, Onoda K (2011). The effects of the behavioral inhibition and activation systems on social inclusion and exclusion. *Journal of Experimental Social Psychology*.

[41] Cope M, Delpy DT, Reynolds EO, Wray S, Wyatt J, van der Zee P (1988). Methods of quantitating cerebral near infrared spectroscopy data. *Advances in Experimental Medicine and Biology*.

[42] Kocsis L, Herman P, Eke A (2006). The modified Beer-Lambert law revisited. *Physics in Medicine and Biology*.

[43] Ye JC, Tak S, Jang KE, Jung J, Jang J (2009). NIRS-SPM: statistical parametric mapping for near-infrared spectroscopy. *NeuroImage*.

[44] Worsley KJ, Friston KJ (1995). Analysis of fMRI time-series revisited—again. *NeuroImage*.

